# HIV-1 Tat Co-Operates with IFN-γ and TNF-α to Increase CXCL10 in Human Astrocytes

**DOI:** 10.1371/journal.pone.0005709

**Published:** 2009-05-28

**Authors:** Rachel Williams, Honghong Yao, Navneet K. Dhillon, Shilpa J. Buch

**Affiliations:** Department of Molecular and Integrative Physiology, University of Kansas Medical Center, Kansas City, Kansas, United States of America; University of Toronto, Canada

## Abstract

HIV-associated neurological disorders (HAND) are estimated to affect 60% of the HIV infected population. HIV-encephalitis (HIVE), the pathological correlate of the most severe form of HAND is often characterized by glial activation, cytokine/chemokine dysregulation, and neuronal damage and loss. However, the severity of HIVE correlates better with glial activation rather than viral load. One of the characteristic features of HIVE is the increased amount of the neurotoxic chemokine, CXCL10. This chemokine can be released from astroglia activated with the pro-inflammatory cytokines IFN-γ and TNF-α, in conjunction with HIV-1 Tat, all of which are elevated in HIVE. In an effort to understand the pathogenesis of HAND, this study was aimed at exploring the regulation of CXCL10 by cellular and viral factors during astrocyte activation. Specifically, the data herein demonstrate that the combined actions of HIV-1 Tat and the pro-inflammatory cytokines, IFN-γ and TNF-α, result in the induction of CXCL10 at both the RNA and protein level. Furthermore, CXCL10 induction was found to be regulated transcriptionally by the activation of the p38, Jnk, and Akt signaling pathways and their downstream transcription factors, NF-κB and STAT-1α. Since CXCL10 levels are linked to disease severity, understanding its regulation could aid in the development of therapeutic intervention strategies for HAND.

## Introduction

Despite the use of combinatorial anti-retroviral therapy (cART) HIV-associated dementia (HAD), a neurological complication in end stage AIDS, still afflicts 9–11% of the HIV infected population [1], [2], [3]. Even more disturbing is the fact that HIV-associated neurocognitive disorders (HAND), which includes HAD, Minor Cognitive Motor Disorders (MCMD), and other HIV related neuropsychiatric impairments, are estimated to affect almost 60% of HIV-1 patients [4], [5]. These patients are diagnosed by changes in behavior, and cognitive and motor abnormalities [5]. HAD, the most severe form of HIV-1 induced CNS impairment [6], is clinically characterized by motor and behavioral dysfunction that in the absence of therapy may lead to seizures, coma, and death within six months of onset [3].

HIV-1 is capable of penetrating the brain shortly after initial infection [1]. However, while cART is able to control the virus in the periphery, the drugs have inferior penetration across the blood brain barrier [7]. So while HIV-1 patients are living longer, they now have to deal with the long term effects of having HIV in the brain. With the increasing prevalence of HAND it is essential to understand the cellular and molecular mechanisms by which HIV exerts its detrimental effects on the CNS. However, since this virus does not infect neurons, the mechanism of neuronal damage and loss seen in HIVE, a pathological correlate of HAD, is not completely understood [8], [9]. Neuronal toxicity is thought to occur, in part, through glial activation and the release of cytotoxic chemokines/cytokines [8], [9], a hallmark feature of HIVE.

Astrocytes are a type of glial cell in the brain capable of releasing neurotoxic chemokines/cytokines after activation by either infection or injury. CXCL10, one of the neurotoxic chemokines released by stimulated astrocytes is up-regulated in the brains and CSF of patients with HIVE and is known to be positively correlated with disease progression [10], [11], [12]. Furthermore, two regulators of CXCL10 expression, IFN-γ and TNF-α, are pro-inflammatory cytokines that are elevated in the brains of patients with HIVE and are also associated with neuropathogenesis [13], [14], [15], [16].

Another positive regulator of CXCL10 induction is the HIV-1 protein, Tat [17], [18], [19]. While astrocytes are not productively infected with HIV-1, the provirus in these cells is able to make the early HIV-1 genes, Tat, Rev and Nef [12], [19]. Tat is not only expressed in astrocytes and other productively infected cells like microglia, but it can also be released from these cells to activate other neighboring cells. Kutsch et. al. (2000) has reported that astrocytes activated with Tat have the ability to release CXCL10. Thus, with the increased expression of Tat and the pro-inflammatory cytokines IFN-γ and TNF-α in brains of patients with HIVE, there exists a perfect milieu for exaggerated induction of CXCL10 and the corresponding neuronal damage.

Increased levels of CXCL10 can be damaging to neurons both directly and indirectly [11], [20], [21]. CXCL10 has direct toxic effects by initiating the activation of a calcium-dependent apoptotic pathway in neurons [11], [20]. Indirectly, CXCL10 has the ability to create a chemotactic gradient between the brain and the periphery, allowing T-cells to infiltrate the brain, a hallmark feature of HAND [8], [21]. This T-cell assault not only weakens the blood brain barrier, but increases local inflammation, which can be damaging to the neurons.

While both cellular (IFN-γ and TNF-α) and viral (Tat) mediators are known to induce CXCL10, it remains unclear how the interplay of these host and viral factors modulates chemokine expression in astrocytes. The data herein demonstrates the increased induction of CXCL10 at both the RNA and protein level in astrocytes activated with HIV-1 Tat, IFN-γ, and TNF-α. The data also reveals that this increase is regulated transcriptionally by the activation of the p38, Jnk, and Akt signaling pathways and their downstream transcription factors, NF-κB and STAT-1α. Since CXCL10 levels are linked to disease severity, understanding its regulation could lead to therapeutic intervention strategies for those suffering from HAND.

## Materials and Methods

### Astrocyte cell culture and treatments

Primary human astrocytes (cat# HA1800; ScienCell Research Laboratories, Carlsbad, CA) were prepared as described by the supplier. The human astrocytic cell lines, U-87 and A172 (ATCC; American Type Culture Collection, Manassas, VA), were grown as described previously [22]. The cells (triplicate or quadruplicate wells) were treated for 6–12 hrs (U87/A172) or 24 hours (primary astrocytes), with: 1) HIV-1 Tat (1–72), 2) a combination of the cytokines IFN-γ (50 ng/ml) and TNF-α (5 ng/ml) or 3) HIV-1 Tat and the cytokines. Control treatments included heat inactivated HIV-1 Tat and cells receiving no treatment. The concentration of Tat in the cerebral spinal fluid (CSF) has been reported at 16 ng/ml [23]. However the concentration of Tat in the brain is unknown, though expected to be much higher than in the CSF [24]. The Tat concentration utilized in this *in vitro* study is generally accepted [25], [26], [27], [28], [29].

The following specific pharmacological inhibitors were used at the final concentration specified: PI3-K Inhibitor LY294002, PLC inhibitor U73122, JAK inhibitor I, JNK inhibitor II, P38 inhibitor SB203580, (all at 10 μM, Calbiochem, Gibbstown, NJ), and NF-κB inhibitor N-*p*-Tosyl-L-phenylalanine chloromethyl ketone (TPCK) (2 μM, Sigma, St. Louis, MO) [30], [31], [32], [33], [34].

### CXCL10 mRNA analysis

RNA was extracted from U-87 astrocytes that were either untreated or treated with HIV-1 Tat and/or the cytokines IFN-γ and TNF-α using TRIzol reagent following the treatment periods (Invitrogen Life Technologies). Quantitative analysis of CXCL10 mRNA was done by quantitative Real-Time PCR using the SYBR Green detection method. RT2 PCR primer pair set for CXCL10 was obtained from SuperArray Bioscience and amplification of CXCL10 from first strand cDNA was performed as described earlier [35] using ABI Prism 7700 sequence detector. Data were normalized using Ct values for the house-keeping gene hypoxanthine-guanine phosphoribosyl transferase (HPRT) in each sample. To calculate relative amounts of CXCL10, the average Ct value of the HPRT was subtracted from that for each target gene to provide changes in Ct value. The fold change in gene expression (differences in changes in Ct value) was then determined as log2 relative units.

### Luciferase assay

To determine the effects of HIV-1 Tat, IFN-γ, and TNF-α on the transcriptional regulation of CXCL10, astrocytes were transfected with either the Luciferase reporter plasmid, TGL-CXCL10 or a GFP plasmid (Amaxa Biosystems, using a Nucleofector kit (Amaxa Biosystems) and allowed to recover overnight [31]. The transfection efficiency of astrocytes was around 30% as determined by analyzing the number of GFP expressing cells in the GFP reporter plasmid transfected wells. Following recovery the astrocytes transfected with the TGL-CXCL10 plasmid were treated with HIV-1 Tat and the cytokines, IFN-γ and TNF-α. After 12 hours the cells were lysed and Luciferase activity was measured using the Luciferase Reporter Assay System (Promega) according to the manufacturer's instructions. The resulting data was normalized by the protein content in each sample. The data represents results obtained from three independent experiments.

### CXCL10 protein analysis by ELISA

Supernatants collected from primary human astrocytes or the astrocytes cell lines U-87 and A172 that were either untreated or treated with HIV-1 Tat and/or cytokines were examined for secreted CXCL10 protein levels using a commercially available CXCL10 ELISA kit (R&D Systems, Minneapolis, MN). The data represents results obtained from three independent experiments.

### Western Blot Analysis

Treated U-87 cells were lysed using the NE-PER Nuclear and Cytoplasmic Extraction kits (Pierce, Rockford, IL). Equal amounts of the corresponding proteins were electrophoresed in a sodium dodecyl sulfate-polyacrylamide gel (12%) in reducing conditions followed by transfer to PVDF membranes. The blots were blocked with 5% non fat dry milk in phosphate buffered saline. Western blots were then probed with antibodies recognizing the phosphorylated forms of Jnk, Akt, p38, (Cell Signaling, Danvers, MA 1:200), STAT1-α (Cell Signaling, 1:500), NF-κB p65 (Cell Signaling, 1:1000), and β-actin (Sigma, St. Louis, MO,1:4000) The secondary antibodies were alkaline phosphatase conjugated to goat anti mouse/rabbit IgG (1:5000). Signals were detected by chemiluminescence (CDP-star; Tropix, Bedford, MA).

### Statistical Analysis

All of the statistical analyses were performed by using a one-tail, independent, Student's *t*-test. The results were judged as statistically significant at *p* values≤0.05.

## Results

### Up-regulation of CXCL10 mRNA in astrocytes treated with Tat, IFN-γ and TNF-α

**Figure 1:**
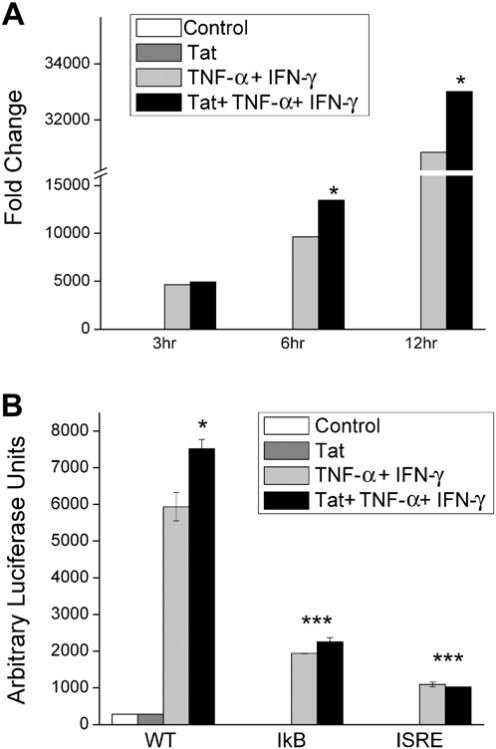
Tat in combination with IFN-γ and TNF-α increases CXCL10 RNA. (A) Real Time PCR analysis showing a significant increase in CXCL10 RNA in U-87 astrocytes treated with a combination of Tat and cytokines. Cells were stimulated with either HIV-1 Tat alone, the cytokines IFN-γ and TNF-α, or Tat and the cytokines together for 6 hour followed by total cell lysis and RNA extraction. (B) Luciferase assay demonstrating the ability of Tat in combination with the cytokines to transcriptionally regulate the CXCL10 gene. The induction of CXCL10 is dependent upon the binding of the ISRE and IκB regulatory sequences. The data represents the mean±SD from three independent experiments (*, p<0.05, ***, p<0.001).

It has been previously shown that HIV-1 in conjunction with the pro-inflammatory cytokines IFN-γ and TNF-α can induce expression of CXCL10 in astrocytes [36]. In this study we wanted to dissect the contribution of HIV-1 Tat in this phenomenon. U-87 astrocytes treated with either the cytokines alone or with Tat plus the cytokines show a significant increase in CXCL10 mRNA as early as three hours by Real Time PCR (Fig. 1A). While Tat by itself did not induce CXCL10 mRNA transcription, when combined with IFN-γ and TNF-α, it was capable of eliciting an increase in CXCL10 mRNA. As shown in Figure 1, cells treated with both Tat and the cytokines for just 6 hours demonstrated an almost 4000 fold increase in CXCL10 mRNA as compared with the cells treated with the cytokines alone, thus underscoring the role of HIV-1 Tat in potentiating the transcriptional regulation of CXCL10.

To confirm the role of Tat in the transcriptional regulation of CXCL10, we performed luciferase reporter assays utilizing the TGL-CXCL10 plasmid [31]. Briefly, U-87 cells were transfected with a TGL-CXCL10 plasmid followed by a 12 hour treatment with HIV-1 Tat and the cytokines, IFN-γ and TNF-α. There was a significant increase in luciferase expression in the cells exposed to both Tat and the cytokines, as compared with cells treated with the cytokines alone, thereby confirming a role for Tat in the transcription of CXCL10 (Fig. 1B). Furthermore, when the U-87 astrocytes were transfected with the TGL-CXCL10 plasmid containing a mutated IFN-stimulated response element (ISRE) or a mutated κB binding site followed by treatment with Tat plus the cytokines, there was little to no luciferase detected above negative control levels. These data thus suggest that Tat in combination with the cytokines can strongly activate the CXCL10 gene, and this activation is highly dependent on the occupancy of the two key regulatory sequences, ISRE and κB.

### CXCL10 protein expression is increased in the presence of Tat and the cytokines

**Figure 2:**
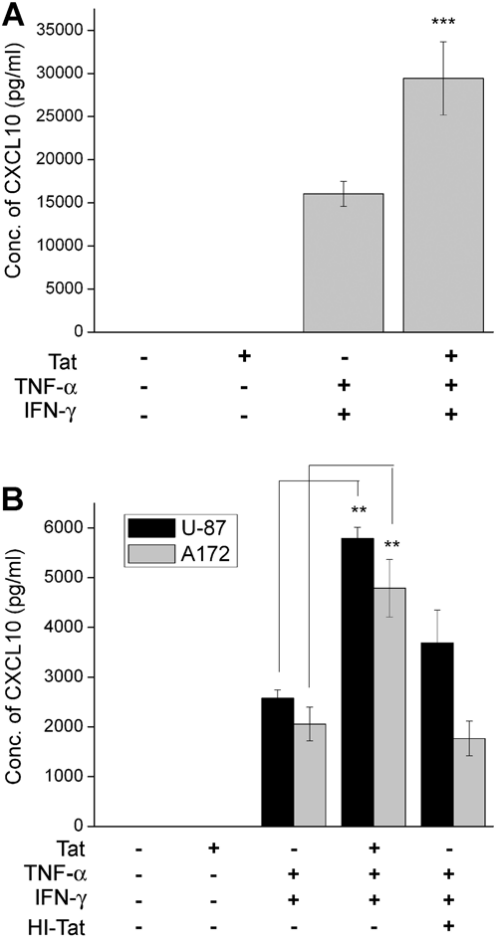
Tat in combination with IFN-γ and TNF-α increases CXCL10 protein. Increased CXCL10 protein expression in (A) primary human astrocytes or (B) U-87 and A172 astrocytes cell lines treated with HIV-1 Tat alone, the cytokines IFN-γ and TNF-α, or Tat and the cytokines together for 24 and 12 hours respectively. Both primary and cell line astrocytes showed a significant increase in CXCL10 protein levels in the cells treated with HIV-1 Tat and the cytokines, than with either treatment alone. Treatment of U-87 and A172 cells with heat inactivated (HI) Tat in conjunction with the cytokines did not lead to an increase in CXCL10 protein levels compared with cells treated with the cytokines alone. The data represents the mean±SD from three independent experiments (**, p<0.01, ***, p<0.001).

To confirm that the increase in CXCL10 RNA correlated with an increase in CXCL10 protein, supernatants from U-87 astrocytes, A172 astrocytes, and primary human astrocytes were collected and analyzed for CXCL10 expression using an ELISA assay. As shown in Figure 2 and similar to the mRNA study, in both the astrocyte cell lines, Tat by itself was unable to induce CXCL10 expression. Similarly to the RNA studies, Tat in combination with IFN-γ and TNF-α, exerted a 2 fold increase in CXCL10 protein levels over that of levels from cells treated with only the cytokine mix. This phenomenon was confirmed in primary human astrocytes and, similar to findings in cell lines, Tat in conjunction with the cytokines resulted in a 2 fold increase in CXCL10 expression over that of the cytokines by themselves. Similar findings in both the cell lines as well as in primary human astrocytes lend credence to the important role of Tat in potentiating cytokine-mediated up-regulation of CXCL10.

### Tat enhances activation of IFN-γ/TNF-α signaling pathways involved in CXCL10 regulation

**Figure 3:**
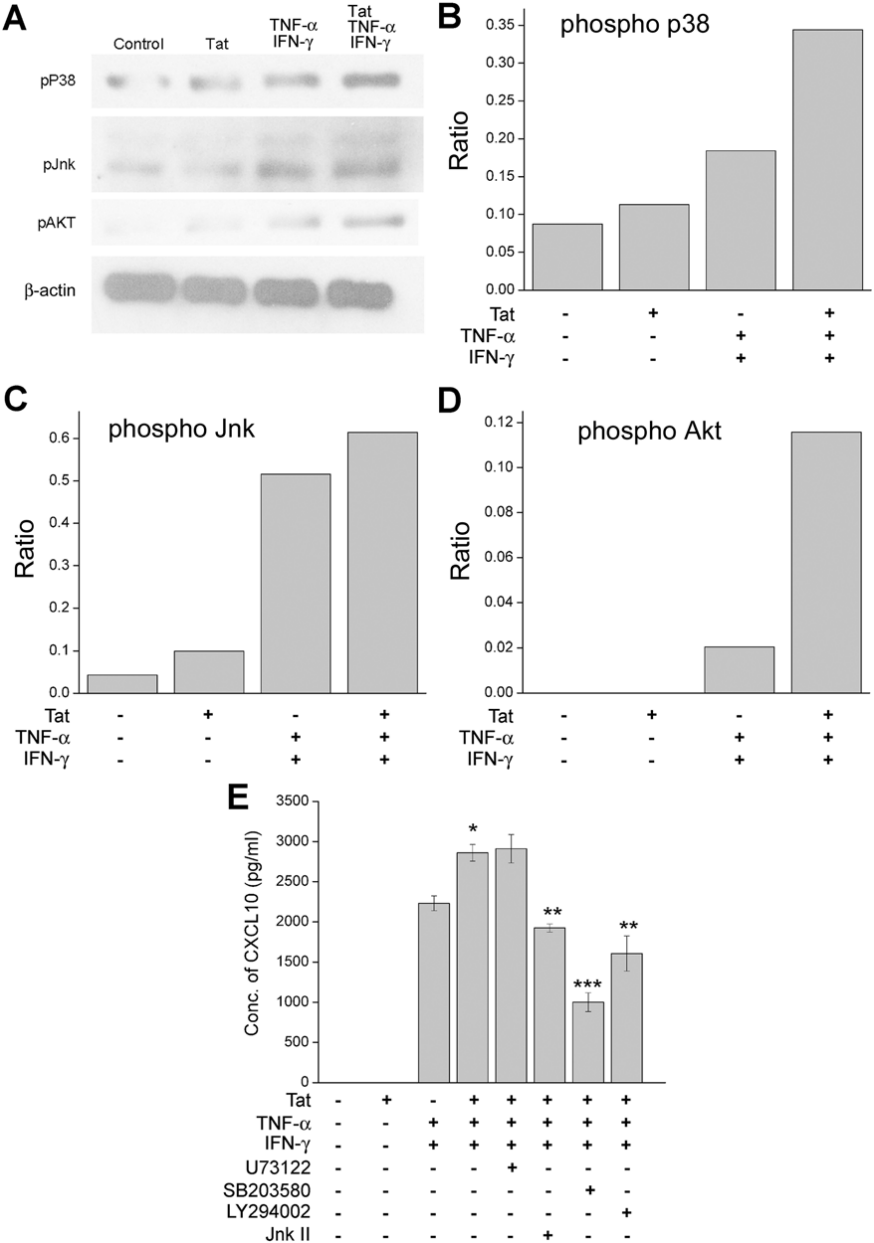
The p38, Jnk, and Akt signaling pathways mediate CXCL10 induction in stimulated U-87 astrocytes. A) Western Blot analysis of cytosolic lysates collected from cells untreated, HIV-1 Tat treated, IFN-γ and TNF-α treated, or treated with Tat in combination with the cytokines for 30 min. The blots were probed with antibodies against phospho-p38, phospho-Jnk, and phosphor-Akt. An antibody against β-actin was used to reprobe the blots for normalization. B), C) and D) Densitometric scans illustrating the ratio of phospho-p38, Jnk, and Akt to β-actin levels. E) Activation of these pathways was shown to be involved in the increased expression of CXCL10 through inhibition of the p38 pathway by SB203580, the Jnk pathway by Jnk II inhibitor, and the Akt pathway by LY294002. Inhibition of the PLC-γ pathway by U73122 had no effect on CXCL10 protein levels. The data represents the mean±SD from three independent experiments (*, p<0.05, **, p<0.01, ***, p<0.001).

Past studies have demonstrated that mitogen activated protein kinase (MAPK) activation is critical in regulating inflammatory responses such as cytokine/chemokine expression in response to multiple stimuli [37], [38], [39], [40]. We next sought to explore whether HIV-1 Tat could potentiate the existing signaling pathways used by IFN-γ and TNF-α. U-87 astrocytes were treated with Tat alone, the cytokines IFN-γ and TNF-α alone, or a mixture of Tat and the cytokines for 30 min. This time point was chosen based on the time-dependent activation of p38, Jnk, and Akt in astrocytes treated with Tat and the cytokines, where 30 min post-treatment was the optimal time for enhanced phosphorylation as determined by Western blot analysis (data not shown). As shown in Figure 3A there was an increase in the activation of Jnk, p38, and Akt in cells treated with the combination of Tat and cytokines compared with cells exposed to either treatment alone. In each instance, as expected each of the cytokine was able to induce phosphorylation of each of the pathways. While Tat was able to mediate only a modest activation of these pathways by itself, in the presence of cytokines, it potentiated a robust activation of these pathways as compared with the cytokines by themselves. These data demonstrate the ability of Tat to enhance signaling pathways activated by other pro-inflammatory molecules, thereby resulting in an increased expression of the target gene CXCL10.

The roles of Jnk, p38, and Akt activation in CXCL10 induction were further examined using a pharmacological approach. U-87 astrocytes were pretreated with either the Jnk II inhibitor, the P38 inhibitor SB203580, or the PI3-K Inhibitor LY294002, all at a concentration of 10 μM, followed by stimulation of cells with Tat and the cytokine mix for 6 hrs (Fig. 3E). Supernatants from treated cells were collected and analyzed for CXCL10 content by ELISA. Pre-treatment of the astrocytes with the Jnk, p38, and Akt inhibitors followed by stimulation with Tat and cytokines resulted in significant reduction of CXCL10, thus underscoring the role of these signaling pathways in the induction of CXCL10.

### Role of transcription factors NF-κB and STAT-1α in induction of CXCL10

The transcription factors NF-κB and STAT-1α play key roles in the induction of CXCL10 [5], [41], [42], [43], [44], [45]. Both of these transcription factors mediate CXCL10 regulation by binding to specific regulatory sequences in the promoter region, the ISRE site for STAT-1α and the κB1 and κB2 sites for NF-κB. Binding of both NF-κB and STAT-1α is necessary for the synergistic increase in CXCL10 mRNA/protein. IFN-γ and TNF-α, through their respective signaling pathways and subsequent activation of NF-κB and STAT-1α, are known to synergistically increase CXCL10 [41], [42]. Therefore, U8-7 cells were treated with either Tat or the cytokines, or Tat in conjunction with the cytokines for 60 min followed by cell lysis and isolation of nuclear proteins. Nuclear extracts were subsequently analyzed for p65-NF-κB and pSTAT-1α content by Western Blot analysis.

**Figure 4:**
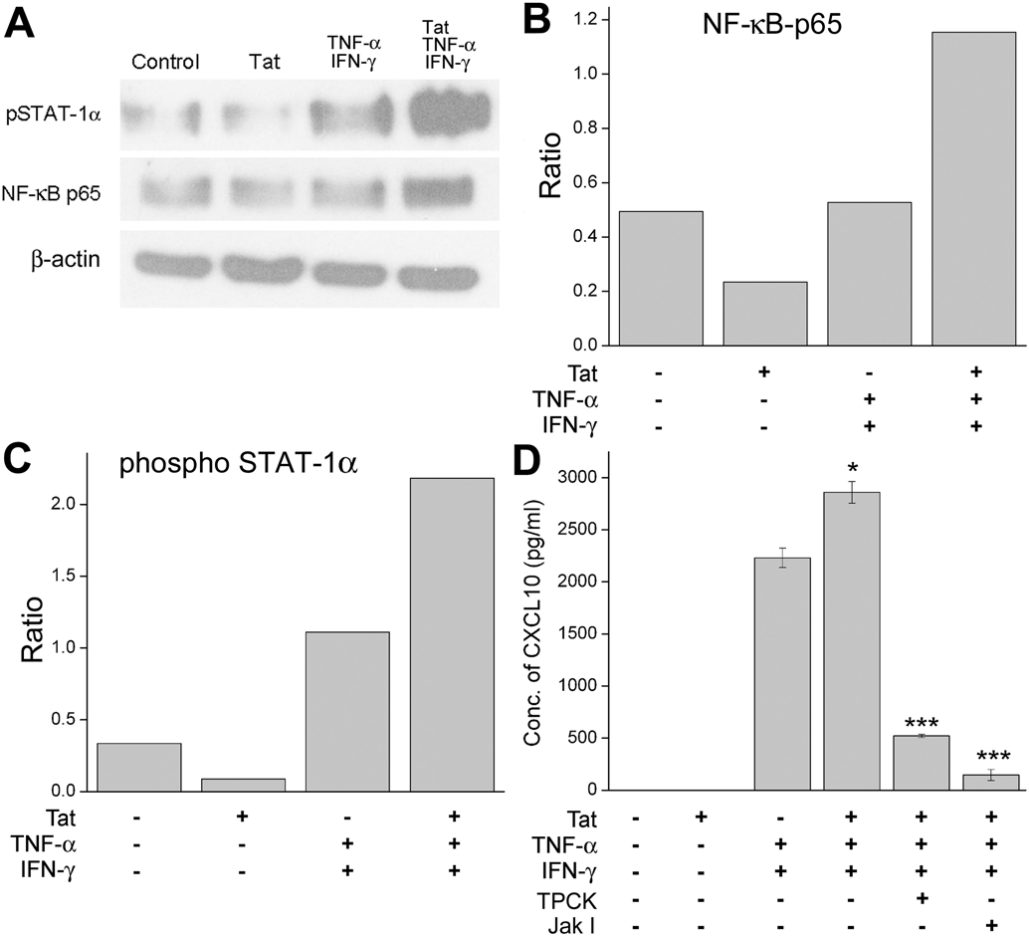
STAT-1α and NF-κB play a role in the increased induction of CXCL10 by HIV-1 Tat and the cytokines in U-87 astrocytes. A) Western Blot analysis of nuclear lysates collected from cells untreated, HIV-1 Tat treated, IFN-γ and TNF-α treated, or treated with Tat in combination with the cytokines for 60 min. The blots were probed with antibodies against phospho-NF-κB p65 and phospho-STAT-1α. Antibodies against β-actin were used to reprobe the blots for normalization. B), C) Densitometric scans illustrating the ratio of phospho-NF-κB p65 and phospho-STAT-1α to β-actin levels. E) Activation of these transcription factors was shown to be involved in the increased expression of CXCL10 through inhibition of the NF-κB by TPCK and the inhibition of the Jak/STAT pathway by a Jak I inhibitor. The data represents the mean±SD from three independent experiments (***, p<0.001).

As demonstrated in Figure 4A the ability of Tat to enhance IFN-γ/TNF-α signaling directly correlates with an increase in the phosphorylation/activation of NF-κB and STAT-1α. To link the increased activation of NF-κB and STAT-1α to CXCL10 regulation, U-87 cells were pretreated with the inhibitors specific for NF-κB (TPCK at 2 μM) or JAK I (JAK I inhibitor at 10 μM) followed by stimulation of cells with Tat and cytokine mix and analyzed for CXCL10 by ELISA. As shown in Fig. 4D inhibition of both NF-κB and STAT-1 resulted in a significant and remarkable reduction in CXCL10 expression in astrocytes, thereby confirming the role of these factors in viral and cytokine-mediated synergistic induction of CXCL10.

## Discussion

Up-regulated expression of chemokines in the brain has been recognized as an important correlate of various neurodegenerative diseases and related neuroinflammation. Chemokines and their receptors are expressed by a wide variety of cells, including those intrinsic to the CNS. These proteins can regulate inflammatory responses by recruiting lymphocytes and monocytes/macrophages to areas of inflammation within the brain contributing to the injury and eventual loss of neurons [46], [47]. Cerebral expression of various chemokines and their receptors is increased in HIVE. CXCL10 was first identified as an early response gene induced after IFN-γ treatment in a variety of cells [48], [49]. Interactions of soluble host factors, such as those between IFN-γ and TNF-α, have been shown to synergistically induce the expression of this chemokine [42]. In addition to its induction by host factors, CXCL10 can also be induced independently by the HIV-1 viral protein Tat [12], [19], [50].

Increased levels of CXCL10 have been detected in the CSF and plasma of individuals with HIV-1 infection [10]. Additionally, brain tissue derived from patients with HAD also reveal increased expression of mRNA for CXCL10 and this expression can be localized to astrocytes [12], [51]. Levels of this neurotoxic chemokine are positively correlated with HAD disease progression [10]. Additionally, up-regulated expression of CXCL10 and its neurotoxic role has also been previously demonstrated in SHIV-infected macaque brains with lentiviral lesions [11], [20].

In the current study we sought to explore the regulation of CXCL10 in human astrocytes stimulated with a mixture of IFN-γ, TNF-α, and HIV-1 Tat. The rationale for using three different stimulants was based on the premise that several host immune and viral factors have the potential to interact during HAD, resulting in neuronal damage and loss. The pro-inflammatory cytokine TNF-α was chosen because it is a key cytokine produced by activated astrocytes and microglia in response to HIV-1 [13], [16]. Levels of this cytokine are also known to positively correlate with HAD pathogenesis [13], [14], [15], [16], [52], [53]. Furthermore, TNF-α has the ability of not only inducing CXCL10 expression by itself [16], [54], but also synergistically with various other host factors, such as IFN-γ, thus increasing the toxicity and inflammation in the surrounding milieu [13], [16], [42], [45], [55], [56].

One of the well-studied host factors that has been shown to co-operatively interact with TNF-α to induce CXCL10 is the pro-inflammatory cytokine IFN-γ [42], [45]. Therefore IFN-γ was also chosen as an additional stimulant in the present study. IFN-γ is a known inducer for the expression CXCL10 in several cell types, including astrocytes [42], [48], [49]. Additionally, IFN-γ has been shown to be markedly increased in CNS tissues during HIV-1 infection in the brain and has been implicated in the pathophysiology of HAD [57].

HIV-1 in combination with IFN-γ and TNF-α has been shown to synergistically up-regulate CXCL10 expression in human astrocytes. However, which viral protein contributes to this effect remains poorly understood. In our attempts to dissect the role of known viral toxins involved in cytokine-mediated induction of CXCL10, we initially examined the effects of HIV-1 Nef, the envelope protein gp120, and Tat in conjunction with the cytokine mix for their ability to up-regulate CXCL10 in U-87 astrocytes. The rationale for selecting these viral toxins stems from their expression in astrocytes and their ability to activate astrocytes [12], [19], [50]. Since HIV-1 Tat (Fig. 2A) but not gp120 or Nef (data not shown) demonstrated the most significant increase with the cytokines in inducing both CXCL10 RNA and protein (Figs 1A and 2A and B), we used HIV-1 Tat for our further studies.

Increased induction of CXCL10 RNA in astrocytes was mediated via transcriptional regulation as demonstrated by transfection of promoter constructs linked to luciferase reporter gene. Using these assays it was demonstrated that combinatorial treatment of astrocytes with the viral and host factors resulted in increased transcription of the luciferase gene. Promoter constructs with mutations in the ISRE (STAT-1α) binding site or IkB (NF-κB) binding sites, the two major regulatory sequences in the CXCL10 promoter [44], [45], [58], however, resulted in abrogation of luciferase expression, thus underscoring the role of each of these binding sites in the induction of CXCL10.

Exploration of the signaling pathways critical for the increased induction of CXCL10 in astrocytes suggested the involvement of the Jnk, p38, and Akt pathways. These findings are in agreement with the synergistic induction of CXCL10 mediated by intact HIV-1 virus and the cytokine mix as reported earlier [36]. In each of the three pathways (Jnk, p38, and Akt) there was modest activation by Tat alone, and definitive activation by the cytokines themselves, however, in the presence of all the three stimulants there was a significant activation of each of these pathways, more so than with either treatment alone. Confirmation of these pathways using specific pharmacological inhibitors further indicated their involvement in the regulation of CXCL10 expression. Activation of the survival factor Akt [59], [60], [61] in the presence of viral and host factors leads us to speculate that this could be a mechanism by the virus to hijack the host machinery in order to maintain a long term reservoir of the neurotoxic inflammatory mediators, including CXCL10. It should be pointed out that inhibition of one specific signaling pathway or transcription factor never completely abrogated the expression of CXCL10, thus indicating involvement of parallel signaling pathways and transcription factors that could be working in unison.

All three pathways (Jnk, p38 and Akt) once activated, can in turn activate the downstream transcription factor NF-κB, which has two separate, yet vital binding sites on the CXCL10 promoter [41]. Multiple studies have demonstrated that astrocytes activated by HIV-1/viral proteins have increased nuclear translocation and activation of the transcription factor NF-κB [36], [62], [63], [64], [65], [66], which, in turn, can regulate CXCL10 expression. In the present study we demonstrate that exposure of astrocytes to Tat and the cytokines results in increased activation and nuclear translocation of the p65 subunit of NF-κB compared with cells treated with either the viral or cellular stimuli. Since Jnk, p38, and Akt all have the ability to activate NF-κB, their convergence on NF-κB could lead to a dramatic increase in CXCL10 induction. In fact, when NF-κB was pharmacologically inhibited, there was a significant inhibition of CXCL10 expression.

While activation of NF-κB can be attributed to all the three stimuli in astrocytes, STAT-1α activation is unique to IFN-γ or IFN-γ plus TNF-α. Intriguingly, our data indicated significant activation of STAT-1α in astrocytes treated with all three stimuli and this was further confirmed using the JAK I specific inhibitor, resulting in a dramatic decrease also in CXCL10 expression. Since Tat does not bind to a known cell receptor, and instead acts by diffusing through the cell membrane, it should be noted that while Tat activation of MAPK signaling pathways and transcription factors has been reported [64], there is a paucity of information of how Tat actually activates these proteins. Recent studies point to the role of Tat-mediated activation of NADPH oxidase, a membrane protein, as a key upstream player involved in the activation of MAPK signaling pathways [67]. It is thus likely that the activation of NADPH oxidase could explain the ability of Tat to activate Jnk, p38, Akt and their downstream transcription factors NF-κB and STAT-1α. Further studies exploring the role of NADPH oxidase in this process are warranted.

**Figure 5:**
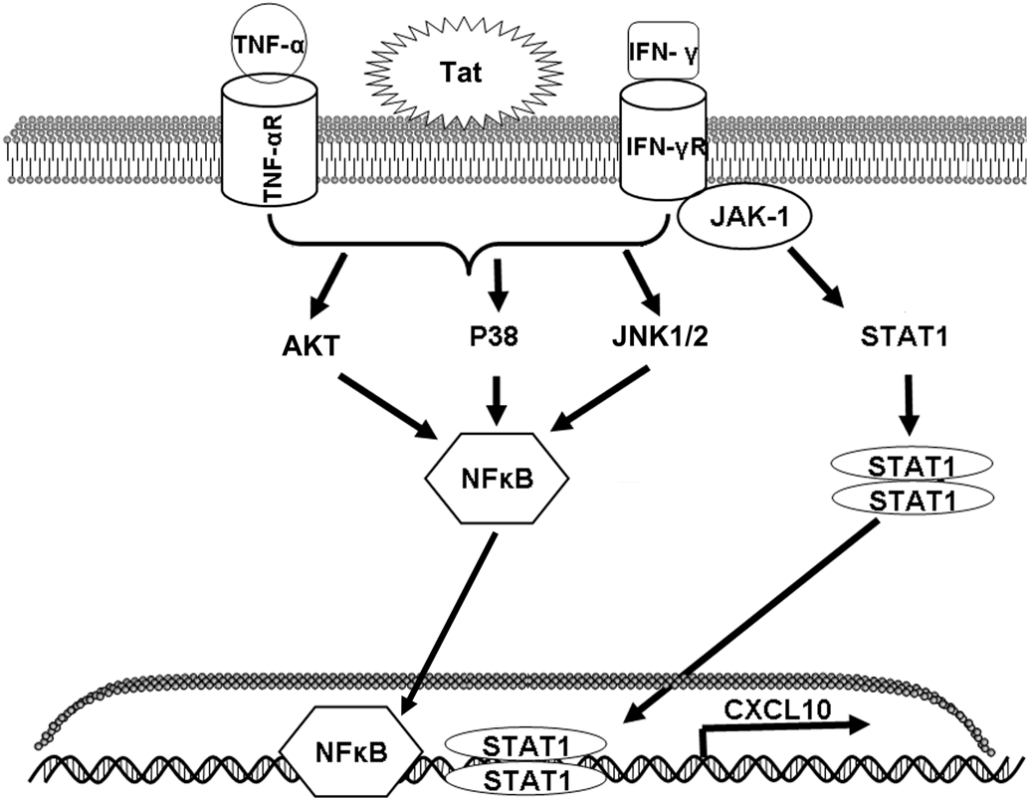
Schematic of the signaling pathways involved in the increased induction of CXCL10 in astrocytes stimulated with HIV-1 Tat in conjunction with IFN-γ and TNF-α. The major signaling pathways activated include p38, Jnk, and Akt, which are able to converge on NF-κB. The activation of NF-κB, along with the activation of STAT-1α, results in the transcription of CXCL10.

In conclusion, we have provided evidence that HIV-1 Tat in conjunction with the cytokines, IFN-γ and TNF-α, is capable of regulating CXCL10 expression in human astrocytes at both the RNA and protein levels. This regulation is likely due to the activation of the Jnk, p38, and Akt signaling pathways and activation of their downstream transcription factors NF-κB and STAT-1 as demonstrated in an overall schematic in Figure 5.

Given that the neuronal toxicity in HAD is thought to occur through glial activation and the release of cytotoxic chemokines/cytokines, dissecting the complex interplay between host factors and viral proteins can lead to a better understanding of disease pathogenesis. The ability of Tat to potentiate activation of signaling pathways stimulated by IFN-γ and TNF-α is an ingenious approach by the virus to exploit the activated cells into generating reservoirs of pro-inflammatory factors, such as CXCL10. This in turn could aid in perpetuating activation, infection, and destruction of several other cell types in the CNS.

Since excessive amounts of CXCL10 can be neurotoxic, our findings lend further credence to the role of CXCL10 in progression of AIDS-associated dementia. The consequences of CXCL10 over expression not only include enhanced influx of inflammatory cells into the CNS, but also amplification of neuronal dysfunction/death in end-stage HAD. Since CXCL10 levels are linked to disease severity, understanding its regulation could aid in the development of therapeutic intervention strategies for HAND.
